# Hydrogel armed with *Bmp2* mRNA-enriched exosomes enhances bone regeneration

**DOI:** 10.1186/s12951-023-01871-w

**Published:** 2023-04-05

**Authors:** Zhujun Yang, Xuejian Li, Xueqi Gan, Mengying Wei, Chunbao Wang, Guodong Yang, Yimin Zhao, Zhuoli Zhu, Zhongshan Wang

**Affiliations:** 1 Department of Stomatology, Xi’an Central Hospital Affiliated to Xi’an Jiaotong University, Xi’an, 710003 Shaanxi China; 2 State Key Laboratory of Military Stomatology & National Clinical Research Center for Oral Diseases & Shaanxi Key Laboratory of Oral Diseases, Department of Prosthodontics, School of Stomatology, Fourth Military Medical University, Xi’an, China; 3 The State Laboratory of Cancer Biology, Department of Biochemistry and Molecular Biology, Fourth Military Medical University, Xi’an, 710032 Shaanxi China; 4 State Key Laboratory of Oral Diseases, National Clinical Research Center for Oral Diseases, West China Hospital of Stomatology, Sichuan University, Sichuan, 610041 Chengdu China; 5 College of Chemistry and Bio-Engineering, Yichun University, Yichun, 336000 Jiangxi China

**Keywords:** Engineered exosome, Gene therapy, *Bmp2* mRNA, Osteogenesis, Hydrogel, CP05

## Abstract

**Background:**

Sustained release of bioactive BMP2 (bone morphogenetic protein-2) is important for bone regeneration, while the intrinsic short half-life of BMP2 at protein level cannot meet the clinical need. In this study, we aimed to design *Bmp2* mRNA-enriched engineered exosomes, which were then loaded into specific hydrogel to achieve sustained release for more efficient and safe bone regeneration.

**Results:**

*Bmp2* mRNA was enriched into exosomes by selective inhibition of translation in donor cells, in which NoBody (non-annotated P-body dissociating polypeptide, a protein that inhibits mRNA translation) and modified engineered BMP2 plasmids were co-transfected. The derived exosomes were named Exo^BMP2+NoBody^. In vitro experiments confirmed that Exo^BMP2+NoBody^ had higher abundance of *Bmp2* mRNA and thus stronger osteogenic induction capacity. When loaded into GelMA hydrogel via ally-L-glycine modified CP05 linker, the exosomes could be slowly released and thus ensure prolonged effect of BMP2 when endocytosed by the recipient cells. In the in vivo calvarial defect model, Exo^BMP2+NoBody^-loaded GelMA displayed great capacity in promoting bone regeneration.

**Conclusions:**

Together, the proposed Exo^BMP2+NoBody^-loaded GelMA can provide an efficient and innovative strategy for bone regeneration.

**Supplementary Information:**

The online version contains supplementary material available at 10.1186/s12951-023-01871-w.

## Background

Bone tissue engineering has progressed over the past few decades. This process traditionally involves seed cells, biomaterials as scaffold components, and growth factors for micro-environments. However, such cell-based therapy still has several drawbacks related to cell source and activity, immunological rejection, long therapeutic duration, and high costs for clinical application. Thus, cell-free approaches, such as those including exosome delivery, gene therapy, or small molecule regulation, have been recently advocated for bone tissue engineering [1–3]. Among these, gene therapy includes both DNA-based and RNA-based treatments to confer the expression of an exogenous gene to change the type, number, and order of nucleotides in parental cells or tissues. This has been extensively explored in the field of regenerative medicine as a safe, effective, and off-the-shelf strategy [4].

In the 1990s, researchers began to use gene therapy for bone defects. Local delivery of BMP2 complementary DNA (cDNA) to the defective bone region continuously produces BMP2 in situ using an endogenous cellular transcription mechanism. The results showed that transgenic cells synthesised BMP2 in the defective bone region and promoted bone healing [5–7]. Unlike DNA therapy, which requires translocation to the nucleus before translation can begin, RNA therapy carries no risk of insertional mutations or other genetic damage [8]. Based on the above principles, gene therapy using mRNA has numerous advantages in treating bone defects over classical gene therapy using DNA [9–11].

Vectors are usually necessary to protect and deliver mRNAs intracellularly and are subject to a short half-life, easy degradation, and inflammation triggered by their interaction with toll-like receptors. Exosomes (Exos) are extracellular vesicles produced by cells with a diameter ranging from 40 to 160 nm (about 100 nm on average) [12]. Donor cells can transport exogenous substances, such as proteins, mRNA, microRNAs (miRNAs), and lipids to recipient cells through exosomes, thereby mediating intercellular communications [13]. Because of their ideal native structure and characteristics, exosomes possess several advantages: an intrinsic homing effect, high stability in circulation, high biocompatibility, low immunogenicity, low toxicity, and effective molecular signalling stimulation. Therefore, exosomes are increasingly employed as a favourable nanoscale carrier for diagnosis and treatment of diseases [14, 15].

In 2020, Zhang et al. [16] found that the exosomes extracted from bone marrow mesenchymal stem cells (BMSC-Exos) activated osteogenic differentiation through the BMP-2/Smad1/RUNX2 signalling pathways. Huang et al. [17] and Li et al. [18] obtained engineered exosomes overexpressing BMP2 by designing the BMP2 plasmid-transfection with bone marrow mesenchymal stem cells (BMSCs), and the results showed that the engineered BMP2-rich exosomes could promote bone healing. However, exosomes containing BMP2 acquired in this manner face problems with low exosome yield and low nucleic acid loading efficiency [19]. Hence, more effective measures are necessary to increase both the exosome yield and the concentration of loaded *Bmp2* mRNAs.

The 5′-end of the mature mRNA undergoes post-transcriptional modification in eukaryotes, namely the m7GPPPN structure, and is also known as the methyl guanosine cap, which is necessary for the initiation of mRNA translation [20, 21]. It provides a signal for the recognition of mRNA by the ribosome, assists ribosomal binding to the mRNA, and enables translation to start from AUG. The cap structure can increase the stability of mRNA and protect mRNA from 5′ → 3′ exonuclease attack [22–24]. D’Lima et al. found a small open reading frame (smORF) in LINC01420. This smORF produces a microprotein named non-annotated P-body dissociating polypeptide (NoBody). NoBody interacts with the mRNA decapping proteins to remove the 5′ cap and promotes the 5′ to 3′ decay [25]. We propose that through the intervention of NoBody, cells excrete translation-inhibited mRNA through exocytosis to form exosomes, thereby increasing the production of exosomes and the expression of the target genes contained therein. Additionally, hydrogel can be utilised as a useful material platform to improve cell status and migration [26, 27]. Hydrogel is a 3D polymer material with high water content and various physical properties. Gao et al. found that phage peptide CP05 (CRHSQMTVTSRL) can capture exosomes of different origins by binding the exosome surface protein CD63 [28].

**Scheme 1:**
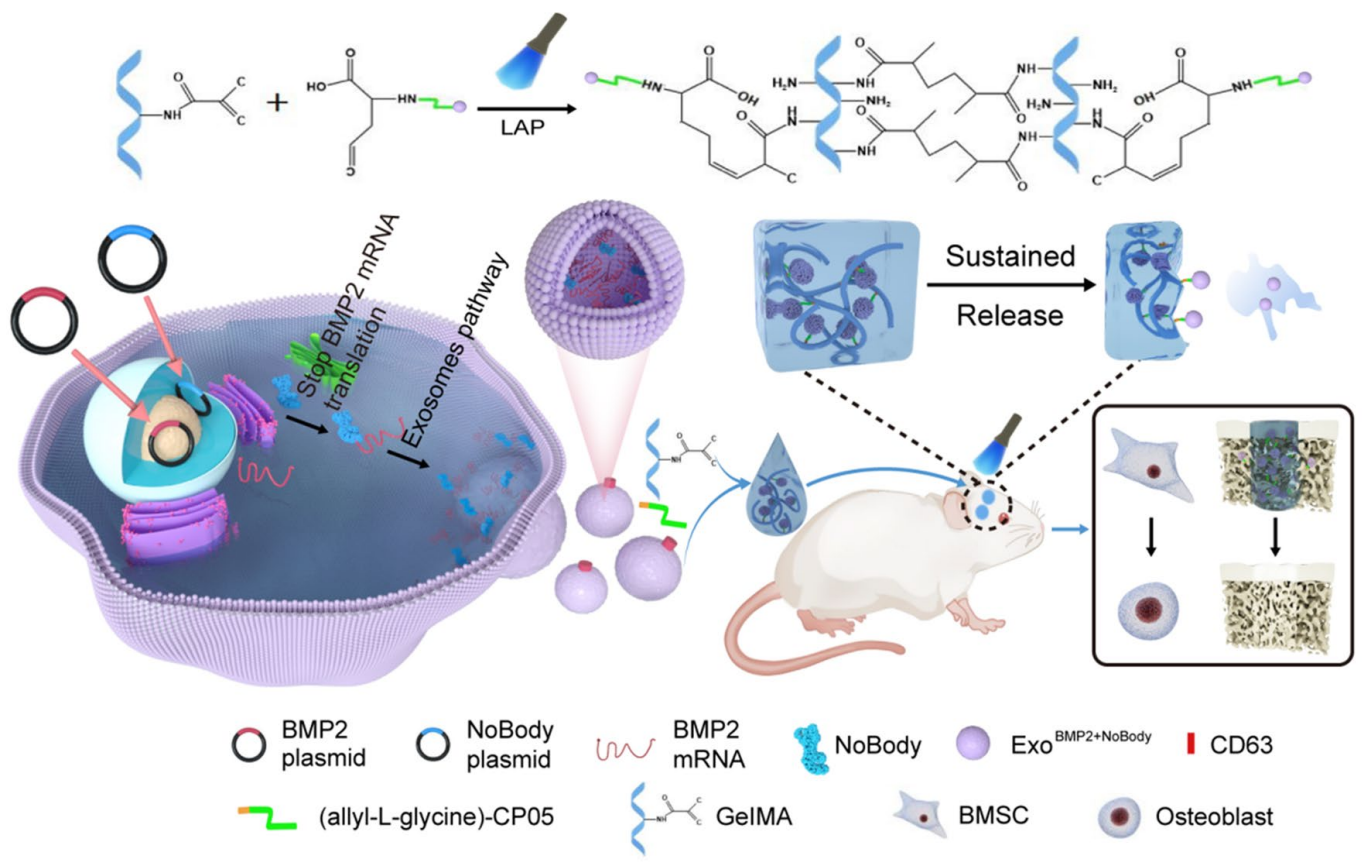
Schematical showing the preparation of Exo^BMP2+NoBody^-loaded GelMA and its effect on bone regeneration

In this experiment, engineered exosomes enriched in *Bmp2* mRNA were obtained by co-transfection of NoBody and Bmp2 artificial plasmids into 293T cells. Allyl-L-glycine-modified CP05 could be covalently bound to GelMA, and GelMA-CP05 was used as a delivery system and scaffold for engineering exosomes to achieve sustained release of engineered exosomes to study the osteogenesis of stem cells in vitro and in vivo. This study provides new strategies for better bone regeneration (see Scheme 1).

## Materials and methods

### Cell preparation and culture

BMSCs were isolated from 8-week-old male Sprague–Dawley (SD) rats by flushing the bone marrow from femurs and tibias with phosphate-buffered saline (PBS; HyClone, USA). BMSCs were cultured in α‐minimum essential medium (Gibco, USA) supplemented with 10% (v/v) foetal bovine serum (FBS; HyClone), 1% (v/v) penicillin/streptomycin (P/S; HyClone). Human embryonic kidney 293T cells (HEK 293T) were purchased from ATCC. HEK 293T were cultured in Dulbecco’s modified Eagle’s medium (DMEM; HyClone) supplemented with 10% (v/v) FBS and 1% (v/v) P/S. All cells were cultured at 37 °C in a humidified atmosphere containing 5% CO_2_/95% air.

### Plasmid construction

Two artificial plasmids (Bmp2 and NoBody) were designed in this experiment based on the properties of NoBody protein to inhibit the mRNA translation process. The sequences of MS2/Linker/intrinsic ribosomal entry site (IRES)/rat BMP2(rBMP2)/Flag/NoBody/MCP are shown in Additional file 1: Table S1. The fragments were cloned into the *NheI* and *BamHI* sites of pcDNA3.1(−) vector to generate the plasmids pCDNA3.1(−)-MS2-Linker-MS2-Linker-IRES-rBMP2 and pCDNA3.1(−)-Flag-NoBody-Linker-MCP.

### Transfection

Two artificial plasmids together (with a molar ratio of 1:1) or two artificial plasmids separately with the empty plasmid pCDNA3.1(−) (with the molar ratio of 1:1) were dissolved in FBS-free DMEM, mixed with Lipofectamine 2000, and incubated at room temperature for 20 min. The plasmids were transfected into 293T cells at 70 − 80% confluence, and the medium was exchanged with fresh 10% FBS-containing medium 6 h later.

### Exosome isolation and characterisation

Exosomes from control or *Bmp2* mRNA-enriched cells were isolated using ultracentrifugation. Briefly, cells were cultured with exosome‐free FBS medium. Cell supernatants were centrifuged at 3000 ×*g* for 30 min to eliminate cellular debris. Next, the supernatant was centrifuged at 100,000 ×*g* for 2 h to obtain the exosomes. After isolation, all the exosomes were resuspended in PBS and stored at − 80 ℃. The size distribution was analyzed by NanoSight (Malvern Instruments Ltd., Malvern, UK). The morphology of isolated exosomes was analyzed by transmission electron microscopy (G2 Spirit Biotwin, TECNAI, USA). In brief, the exosomes were applied on a carbon copper grid and air dried for 2 min. Next, they were rinsed with deionized water for 1 min and stained with 2% uranyl acetate for 30 s. The images were analyzed by a transmission electron microscope. To investigate the characteristics of intracellular internalization of exosomes, cells and DiI-labelled exosomes were co-cultured for 6 h, washed three times in PBS, and the rat bone marrow mesenchymal stem cells (rBMSCs) were fixed in 4% paraformaldehyde for 15 min and washed again. Cell nuclei were stained with Hoechst for 10 min. The cellular distribution of the exosomes was imaged using a confocal laser scanning microscope (Nikon A1R, Tokyo, Japan).

### qRT-PCR

The total RNA from exosomes was extracted using TRIzol reagent (Invitrogen, USA), according to the manufacturer’s protocol. Reverse transcription was performed using the PrimeScript First-Strand cDNA Synthesis Kit (Takara, China) for analysis of mRNA expression. Subsequently, qPCR reactions (20 µL) were performed using FastStart Essential DNA Green Master. *GAPDH* was used as an internal control to normalise signal for each target gene. Relative expression was calculated by the 2^–ΔΔCt^ method. The sequences of PCR primers for *BMP2*, *OPN*, *COL1a*, *ALP,* and *GAPDH* used in this study are listed in Additional file 1: Table S2.

### Western blot assay

Total protein from the donor cells or isolated exosomes was extracted with RIPA Lysis Buffer (Beyotime, China) at 4 ℃ for 30 min. Protein concentration was determined using the BCA Protein Assay Kit (Pierce, USA) and proteins were separated using SDS-PAGE with a 6% stacking gel and 12% resolving gel. The proteins were then transferred to nitrocellulose membranes. After being blocked with 3% bovine serum albumin, the membranes were incubated with primary antibodies against BMP2 (pa5-69,363, ThermoFisher), FLAG (ab205606, Abcam), GM130 (11,308–1-AP, ProteinTech), TSG101 (ab83, Abcam), CD81 (ab286173, Abcam), Hsp70 (4872T, Cell-Signaling), OPN (sc-73631, Santa Cruz Biotechnology), COL-1 (ab260043, Abcam) and anti-GAPDH (D110016-0100, BBI Life Sciences) at 4 ℃ for 12 h. After being washed three times in TBST, the membranes were incubated with secondary antibodies (anti-mouse [7076, CST] or anti-rabbit [7074, CST]) in Tris-buffered saline at room temperature for 1 h. The images were developed by chemiluminescence (GE Healthcare, Chalfont St. Giles, UK) in a dark room.

### Materials

Gelatin methacryloyl (GM-90, GM-60) and lithium pherryl-2,4,5-trimethylbenzoylphosphinate (LAP) were purchased from Engineering for Life (Suzhou, China). FITC-labelled CP05 and FITC-labelled (allyl-L-glycine)-CP05 were purchased from Bankpeptide (Hefei, China).

### Slow-release effect of a hydrogel encapsulating exosomes

GelMA (EFL, EFL-GM-60, Suzhou, China) and GelMA (EFL, EFL-GM-90, Suzhou, China) were dissolved in 0.5% (w/v) PBS. The solution was treated with 0.5 wt% at visible light (405 nm), and the mixture was incubated at 60 °C for approximately 30 min to completely dissolve the solids. The solution was filter-sterilised using a 0.22 µm filter. Modified hydrogel solutions were prepared by dissolving CP05 or (allyl-L-glycine)-CP05 [0.1% (w/v)], and exosome [20% (w/v)] in GelMA solution [10% (w/v)] at 37 °C. (Fig. 3A).

Each hydrogel sample was incubated in PBS at 37 °C under constant shaking at 100 rotations/min (r/min) to mimic dynamic flow of blood and lymph in the body as described before [29]. Specimens (GM-60, GM-90, GM-60 + Exo, GM-90 + Exo, GM-60/CP05 + Exo, GM-90/CP05 + Exo, GM-60-CP05 + Exo, and GM-90-CP05 + Exo, n = 3/group) were weighed at baseline (W0), which represented 0 h. Samples were renewed with fresh solution every 10 h. At the specific time points (10, 20, 30, 40, 50, and 60 h), the specimens were removed from the solution, washed twice with sterile deionized water, drained through filter paper, and reweighed (Wt). The remaining mass ratio (%) of each hydrogel sample was calculated as follows: remaining mass ratio = (Wt/W0) × 100%. In the GM-60(90)/CP05 + Exo group, the exosomes were not covalently linked to the hydrogel; however, in the GM-60(90)-CP05 + Exo group, the exosomes were covalently linked to the hydrogel. Among them, 500 µL of GM-90-CP05 + Exo hydrogel was injected subcutaneously under the middle dorsal regions of the rat skin to investigate the degradation rates of the experimental group in vivo. At the specific time points (0, 2, 4, 6, 8, and 10 d), the specimens were removed from the rat, drained through filter paper, and reweighed (Wt). The weight residual (%) of each hydrogel sample was calculated as follows: weight residual = (Wt/W0) × 100%. The experimental animal procedure was conducted under strict supervision and approved by the Animal Care and Use Committee of Fourth Military Medical University.

DiI-labelled exosomes (500 µg/mL) were loaded into control and experimental groups of hydrogel and incubated in 1 mL PBS at 37 °C at 100 r/min to monitor exosomes. Cumulative exosome release was monitored by removing and replacing the buffer every 10 h and exosome-associated fluorescence was analyzed by confocal laser scanning microscope (Nikon A1R, Tokyo, Japan). The exosome concentration was detected in PBS prior to each change using the ELISA kit (Elabscience, China).

Each sample was placed at − 80 °C overnight and then taken out and placed on a silicon wafer and freeze dried (GOLD-SIM, US) at − 80 °C. Samples were examined using a scanning electron microscope (S-4800, Hitachi, Japan) at an accelerating voltage of 5 kV. The samples were loaded on top of the conductive tape and sputter-coated with gold for 60 s with a magnetron sputtering apparatus (E-1045, Hitachi).

### Evaluation of the biocompatibility between cells and hydrogel in vitro

Hydrogel (1 mL) was injected into the confocal dish and irradiated with light at 405 nm for 10 s. rBMSCs were seeded on the cured hydrogel and cultured for 1 and 3 days. The cells were rinsed three times with PBS solution and incubated with Calcein/PI Cell Viability/Cytotoxicity Assy Kit (Beyotime) for 30 min at 37 ℃. The cells were then rinsed three times with PBS solution and observed using a confocal laser scanning microscope (Nikon A1R, Tokyo, Japan) and analysed by Image J version 1.48 (USA).

Hydrogel (100 µL) was injected into 96-well culture plates and irradiated with light at 405 nm for 10 s. BMSCs were seeded on the cured hydrogel and cultured for 1, 3, 5, and 7 days. The proliferation of rBMSCs seeded on hydrogel was measured with the CCK8 Assay Kit (Beyotime) and a microplate reader (Tecan, Spark 20 M, Shanghai, China) at 450 nm. Each group was tested in triplicate.

### Cellular uptake and intracellular internalization of exosomes

To further explore the exosome uptake action by cells on the hydrogel, rBMSCs were seeded onto hydrogel conjugated with DiI-labelled exosomes and incubated for 48 h. After washing three times in PBS, the rBMSCs were fixed in 4% paraformaldehyde for 15 min and then washed again. Cell nuclei were stained with Hoechst for 10 min. The cellular distribution of the exosomes was imaged using a confocal laser scanning microscope (Nikon A1R).

### ALP stain, ALP activity, Alizarin red stain, and qualification

For investigating the extent of osteogenic differentiation in the 2D and 3D environment, BMSCs with and without hydrogel were cultured in osteogenic medium without exosomes for a fixed time interval. To measure ALP expression, cells were fixed with 10% formalin, stained using BCIP/NBT ALP Colour Development Kit (Beyotime), and imaged with an inverted fluorescence microscope (DMI6000 B, Leica, Germany). To quantitate ALP activity, cells were tested with an ALP Assay Kit (Beyotime). Absorbance was measured at 405 nm. To investigate mineral deposition, cells were fixed with formalin and stained with Alizarin Red S Staining Kit for Osteogenesis (Beyotime) for approximately 20 min, washed three times with dH_2_O and stained with ARS for 20 min at room temperature. Cells were imaged with a Leica MDI6000 B fluorescence microscope. After several washes with dH_2_O, the stain was desorbed with 200 µL 10% cetylpyridinium chloride (Sigma, Germany) for 1 h. The dye was collected, and the absorbance was read at 590 nm using a spectrophotometer.

### Establishment of rat calvarial defect model

The experimental animal procedure was conducted under strict supervision and approved by the Animal Care and Use Committee of Fourth Military Medical University. A total of 72 eight-week-old male SD rats were used in this study. The rats were randomly divided into NC, GM90, GM90 + Exo^None^, GM90-CP05 + Exo^None^, GM90 + Exo^BMP2^, and GM90-CP05 + Exo^BMP2^ (n = 6/group). Rats were anesthetized with pentobarbital sodium (50 mg/kg). Bilateral critical full-thickness cranial defects (d: 5.0 mm) were drilled using a trephine drill. Hydrogel (100 µL) was then injected into each defective region, layered, and closed with 5–0 absorbable sutures. Drinking water containing trimethoprim-sulfamethoxazole was provided for 7 days to prevent infections. The rats were euthanized at weeks 4 and 8 post-surgery, and the samples were collected and fixed for analysis.

### Micro-CT analysis

To evaluate bone regeneration in the defective area, the harvested calvarias were scanned by high-resolution Micro-CT (Siemens, Inveon MM Micro CT, USA). Samples were reconstructed and analyzed using supporting software (Inveon Research Workplace 2.2). The various bone parameters, including BS/TV (%), Tb. Sp (mm), and Tb. T (mm), were analyzed.

### Histology, immunohistochemistry, and immunofluorescence analyses

The samples were decalcified in 10% disodium ethylenediaminetetraacetic acid (Solarbio, China) at 4 °C for 30 d. Afterwards, decalcified samples were longitudinally embedded in paraffin wax and sliced into 5 μm sections. Then, the tissue sections were stained with haematoxylin and eosin (Solarbio) and TRAP staining kit (Solarbio) based on the manufacturer’s instructions. The stained sections were photographed under the microscope (DM6000 B, Leica) and analyzed with Image ProPlus 6.0 Software (Media Cybernetics, Silver Spring, USA). OCN (PB1009, Boster) and COL-1a (PB0981, Boster) staining was performed with an anti-rabbit HRP/DAB Detection Kit (18653 s, CST), following the manufacturer’s protocol. The images were photographed under the microscope. Semiquantitative analysis was measured by Image Proplus 6.0 software. For histology evaluation, 3 experienced orthopedical pathologists were invited.

### Statistical analysis

Data are expressed as the mean ± SEM and were analyzed by one-way ANOVA for a three-group comparison and log rank test for survival curves. Differences were considered significant at P < 0.05. All statistical analyses were conducted with GraphPad Prism 8.0.

## Results

### Characterization of the engineered exosomes enriched in *Bmp2* mRNA

**Fig. 1:**
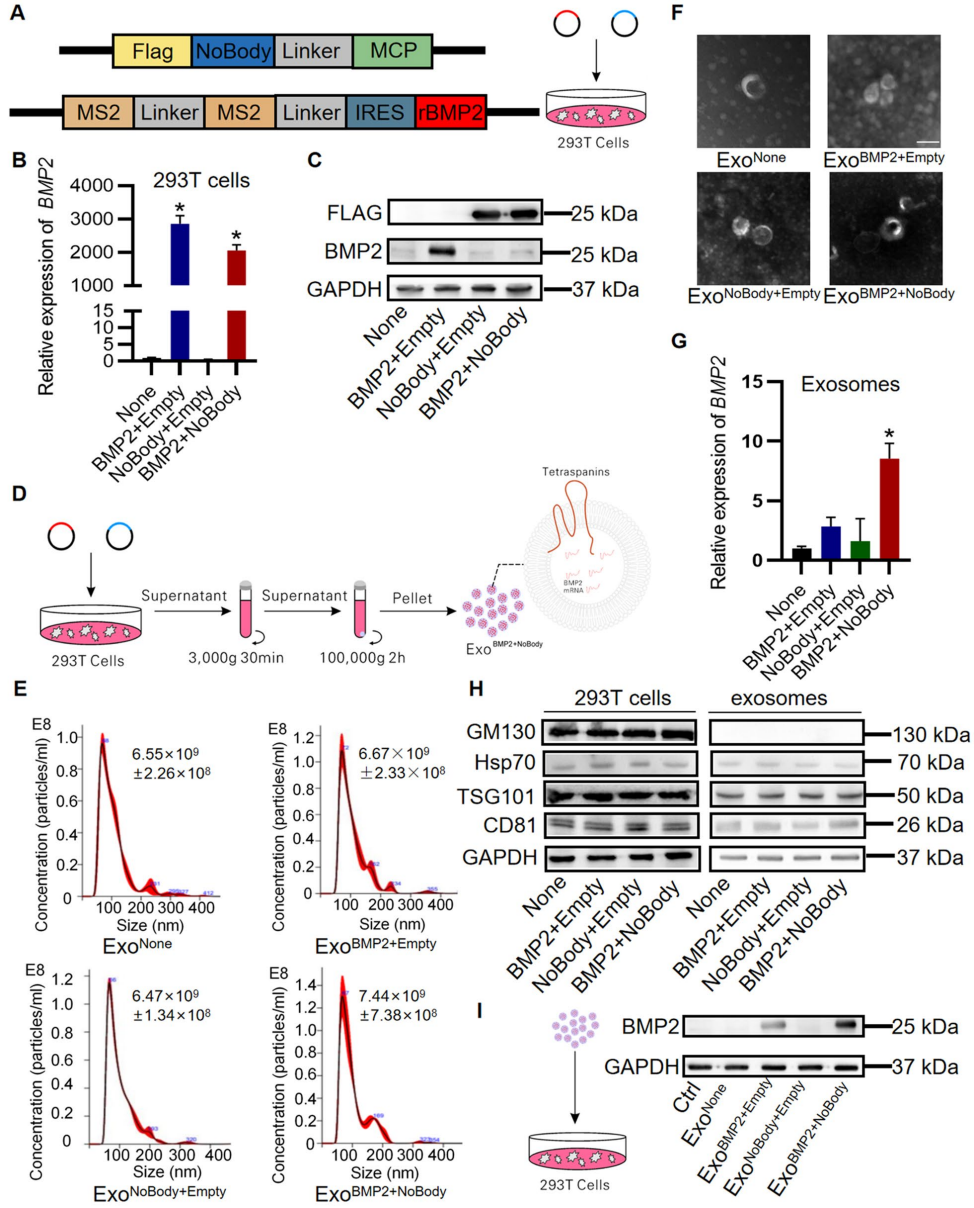
Characterisation of the engineered exosomes enriched for *Bmp2* mRNA. **A** Design of artificial plasmids and selection of restriction endonuclease sites. **B** 293T cells-expressed BMP2 genes were measured using qRT-PCR assay. **C** Western blot analysis of the expression of BMP2 in the parental cells. GAPDH served as the loading control. **D** 293T cells as exosome donor cells were transfected with the above artificial plasmids, followed by engineering exosome isolation and characterization. **E** Size distribution of the control and engineering exosomes were analysed using NanoSight. **F** Representative transmission electron microscope image of the control and engineering exosomes. Scale bar = 100 nm. **G** Exosomes-expressed *Bmp2* genes were measured using qRT-PCR assay. **H** Western blot analysis of the expression of BMP2 and exosomal markers in both the parental cells and derived exosomes. GAPDH served as the loading control. **I** Western blot analysis of 293T cell BMP2 expression after co-culture of exosomes with 293T cell. GAPDH served as the loading control

To develop engineered exosomes enriched in *Bmp2 mRNA*, two artificial plasmids (BMP2 and NoBody) were co-transfected into 293T cells (Fig. 1A). qPCR and western blot analysis confirmed that donor cells expressed the NoBody protein. The NoBody protein inhibited the expression of BMP2 protein in donor cells, *Bmp2* mRNA expression in experimental group was slightly inhibited (Fig. 1B, C). And the cells cultured in exosome-free FBS medium to obtain engineered exosomes, named Exo^BMP2+NoBody^ (Fig. 1D). The exosomes obtained from non-transfected plasmids or cells transfected with a single artificial plasmid were used as controls and named Exo^None^, Exo^BMP2+Empty^, and Exo^NoBody+Empty^, respectively. Nanoparticle tracking analysis and transmission electron microscopy further confirmed that the isolated extracellular vesicles conformed to exosome size and morphology (Fig. 1E, F). Western blot analysis showed that the isolated extracellular vesicles had exosomal properties in both the experimental and control groups (Fig. 1H). In exosomes from 293T cells, *Bmp2* mRNA expression in Exo^BMP2+NoBody^ was eight-fold higher than that in the blank group, and approximately three-fold higher than that in the Exo^BMP2+Empty^ group (P < 0.05). (Fig. 1G). The results of 293T cells co-cultured with Exo^BMP2+NoBody^ indicated that 293T cells internalise Bmp2 mRNA-enriched exosomes and highly expressed BMP2 protein. Consistent with up-regulated expression of *Bmp2* mRNA in the experimental group vs the control group, increased BMP2 protein expression was also detected. This indicates that the NoBody protein interacting with *Bmp2* mRNA has no inhibitory effect on *Bmp2* mRNA translation after entering the recipient cell through the exosome pathway (Fig. 1I).

### Enhanced osteogenesis mediated by Exo^BMP2+NoBody^

**Fig. 2:**
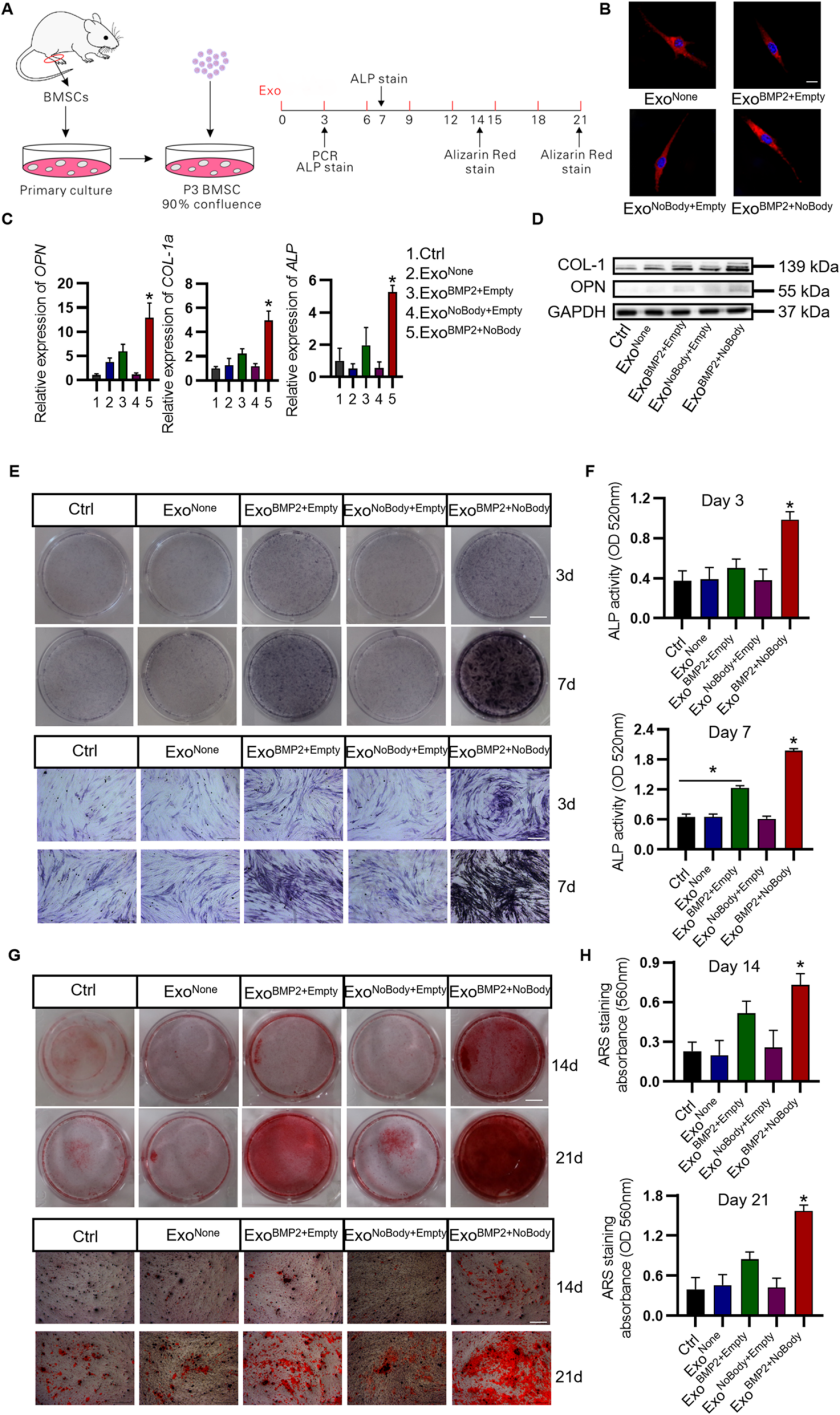
Enhanced osteogenesis mediated by Exo^BMP2+NoBody^. **A** Schematic representation of the exosome treatment. Time interval of exosome injection and detection index of in vitro Exo&BMSC co-culture. **B** Internalisation of DiI-labelled EXOs into BMSCs was visualized using confocal images. Scale bar = 10 μm. **C** Expression of osteogenic related genes (*OPN, ALP* and *COL-1a*) was measured using qRT-PCR assay on day 3. **D** Expression of osteogenic related protein (*OPN* and *COL-1*) was measured using western blot on day 7. *ALP* expression (using ALP staining) (**E**) and activity (**F**) were assessed on days 3 and 7, respectively. Scale bar = 1 cm (up); Scale bar = 500 µm (below). Mineralization of BMSCs was detected using Alizarin red staining (**G**) and quantified (**H**) on days 14 and 21, respectively. Scale bar = 1 cm (up); Scale bar = 500 µm (below)

To investigate whether the engineered exosomes can promote the osteogenic effect of stem cells in vitro, rBMSCs were extracted from rat femurs and tibias and co-cultured with the engineered exosomes (Fig. 2A). Confocal imaging revealed that all groups of exosomes exhibited similar biological internalization and efficient transfer to target cells (Fig. 2B). After 3 days of co-culture, it was detected by RT-qPCR. Compared with that in Exo^BMP2+Empty^, Exo^NoBody+Empty^, or control exosome-treated cells, the expression of major osteogenic genes such as *ALP*, *OPN,* and *COL1a* was significantly increased in Exo^BMP2+NoBody^-treated cells (P＜ 0.05) (Fig. 2C). After 7 days of co-culture, the expression of related osteogenic proteins such as *OPN* and *COL1* were significantly increased in EXO^BMP2+NoBody^-treated cells (Fig. 2D). Furthermore, increased osteogenic differentiation stimulated by Exo^BMP2+NoBody^ and ALP expression and mineralization levels were increased, as detected by ALP staining/activity and Alizarin red staining/quantification (Fig. 2E-H).

### Modification and properties of the GM-90-CP05 + Exo hydrogel

**Fig. 3:**
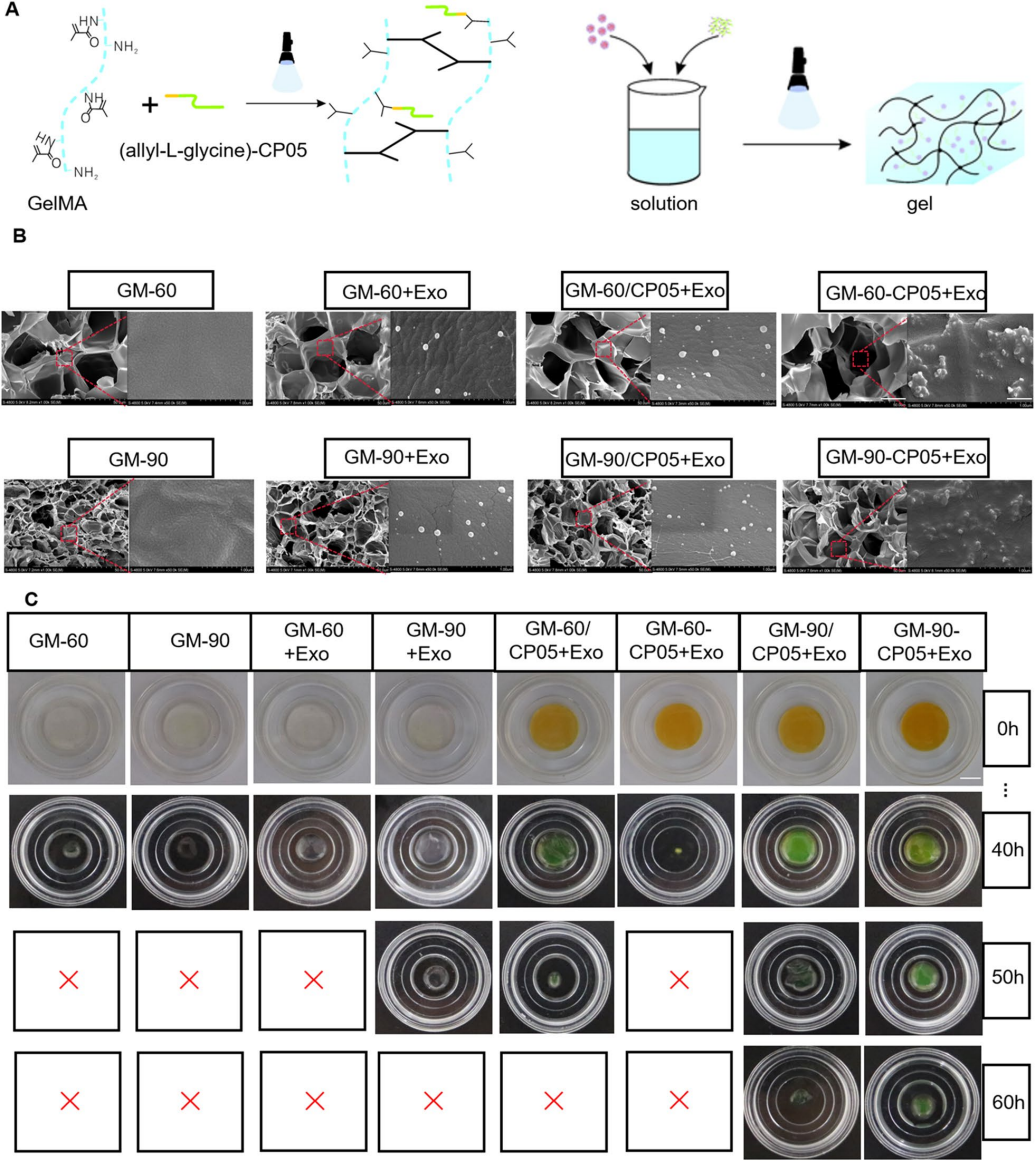
GelMA-CP05 + Exo hydrogel prolonged hydrogel degradation and exosome release. **A** The chemical molecular structure of the allyl-L-glycine CP05 polypeptide and GelMA, and the structure of GelMA-CP05 under visible light (405 nm) are shown. **B** SEM images of the six types of hydrogel (GM-60, GM-90, GM-60 + Exo, GM-90 + Exo, GM-60/CP05 + Exo, GM90/CP05 + Exo, GM-60-CP05 + Exo, and GM-90-CP05 + Exo). Scale bar = 25 µm (left); Scale bar = 500 nm (right). **C** General photographs of the hydrogel from the degradation experiments were taken at 0, 40, 50, and 60 h. Scale bar = 1 cm

**Fig. 4:**
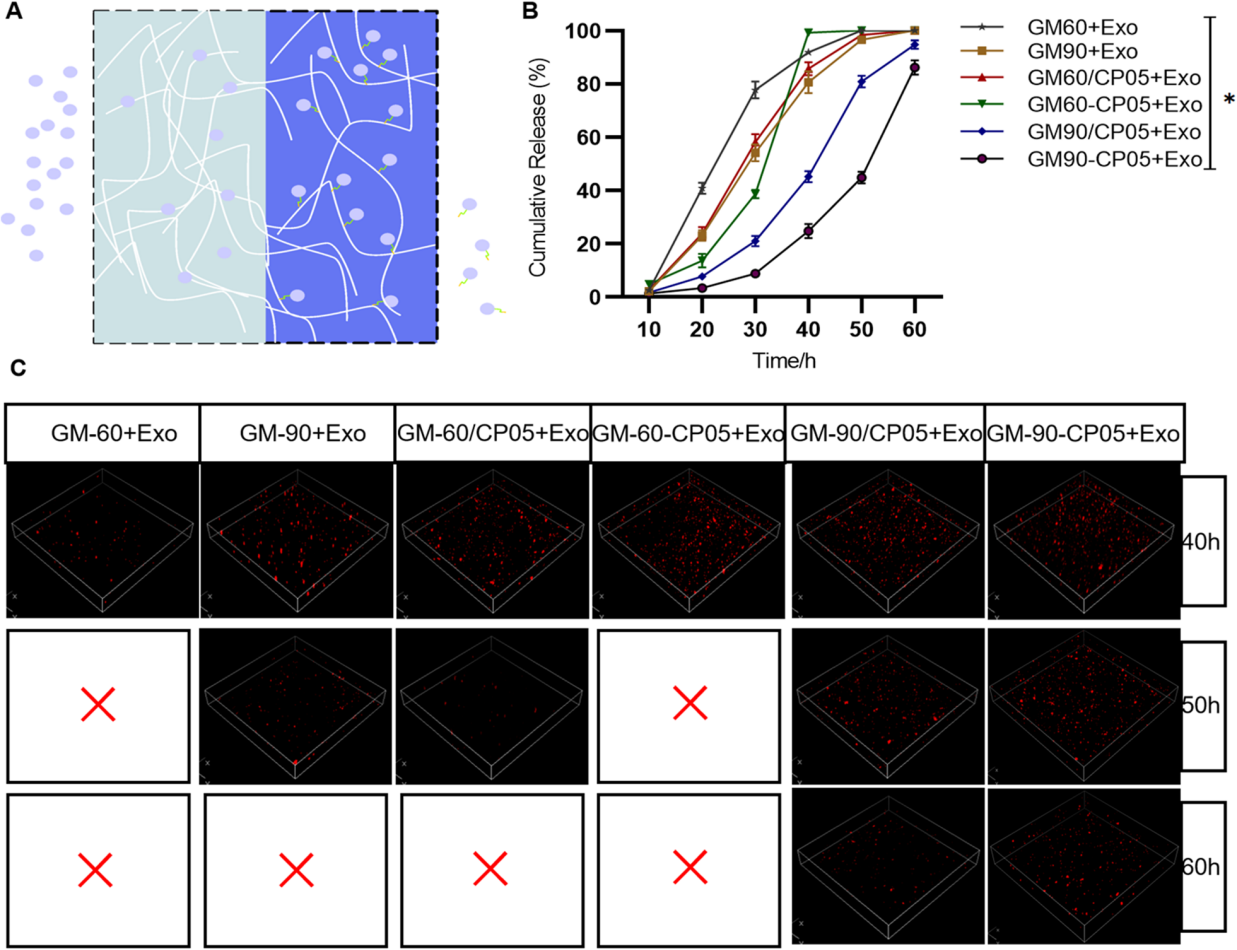
Sustained release effect of the GelMA-CP05 + Exo hydrogel. **A** Schematic diagram of the exosome sustained release by the modified hydrogel. **B** Exosome release by the hydrogel detected at 6-h intervals. **C** Retention rate of DiI-labelled EXOs in the hydrogels was visualised using 3D confocal images at 40, 50, and 60 h

GelMA has been widely used in various biomedical applications, especially bone regeneration and angiogenesis, because of its biocompatibility, biodegradability, strong hydrophilicity, high side chain reactivity, and stable physicochemical properties [30]. Good tissue engineering scaffolds require appropriately sized and continuous voids, which facilitate sustained exosome release, cell growth, and nutrient cycling. Allyl-L-glycine modified CP05 can undergo an additional reaction with the side chain of GelMA to form a covalent bond (Additional file 1: Fig. S1). After lyophilisation, SEM showed that the different groups had porous connecting structures. The pore size of GM-90 was significantly smaller than that of GM-60, the pore size and structure of GelMA was not affected by addition of exosomes or CP05 without allylglycine modification. However, the addition of allylglycine-modified CP05 increased the original pore size through an additional reaction with the GelMA side chain. Exosomes in the GM-90-CP05 and GM-60-CP05 groups were more enriched than those in the other groups, and the binding mode between exosome and hydrogel included not only simple adhesion and encapsulation, but also covalent binding mediated by modified CP05 (Fig. 3B). To detect degradation of the hydrogels, we immersed each set of 10% of the hydrogels in PBS at 37 °C for shaking at a constant speed of 100 r/min and measured the remaining mass every 6 h. We found that GM-60-CP05 + Exo hydrogel had the fastest degradation rate, and GM-90-CP05 + Exo hydrogel had the slowest degradation rate (Fig. 3C, Additional file 1: Fig. S2). GM-90-CP05 + Exo degradation curve trend in vivo was similar to the in vitro experiments (Additional file 1: Fig. S7). To detect the exosome release of the hydrogel, DiI-labelled exosomes were mixed into the hydrogel solution at 500 µg/mL. After 405 nm wavelength illumination, 1 mL of PBS was added to the container and shaken at 100 r/min at 37 °C. PBS was aspirated every 6 h, and new PBS was added. Confocal laser images and CD63 ELISA showed that exosomes in GM-90-CP05 were released for the longest duration when compared with exosomes in GM-90-CP05, and exosomes in GM-90/CP05 were released faster. However, GM-60 + Exo exosomes were released at the fastest rate because of the fastest degradation (Fig. 4B, C; Additional file 1: Fig. S5). The fluorescence intensity curve also matched the above results (Additional file 1: Fig. S4). In conclusion, GM-90-CP05 is a hydrogel that can degrade slowly and prolong the release time of exosomes. We chose GM-90-CP05 as the engineered exosome delivery carrier.

### GM-90-CP05 hydrogel with encapsulation of Exo^BMP2+NoBody^ enhances osteogenesis in vitro

**Fig. 5:**
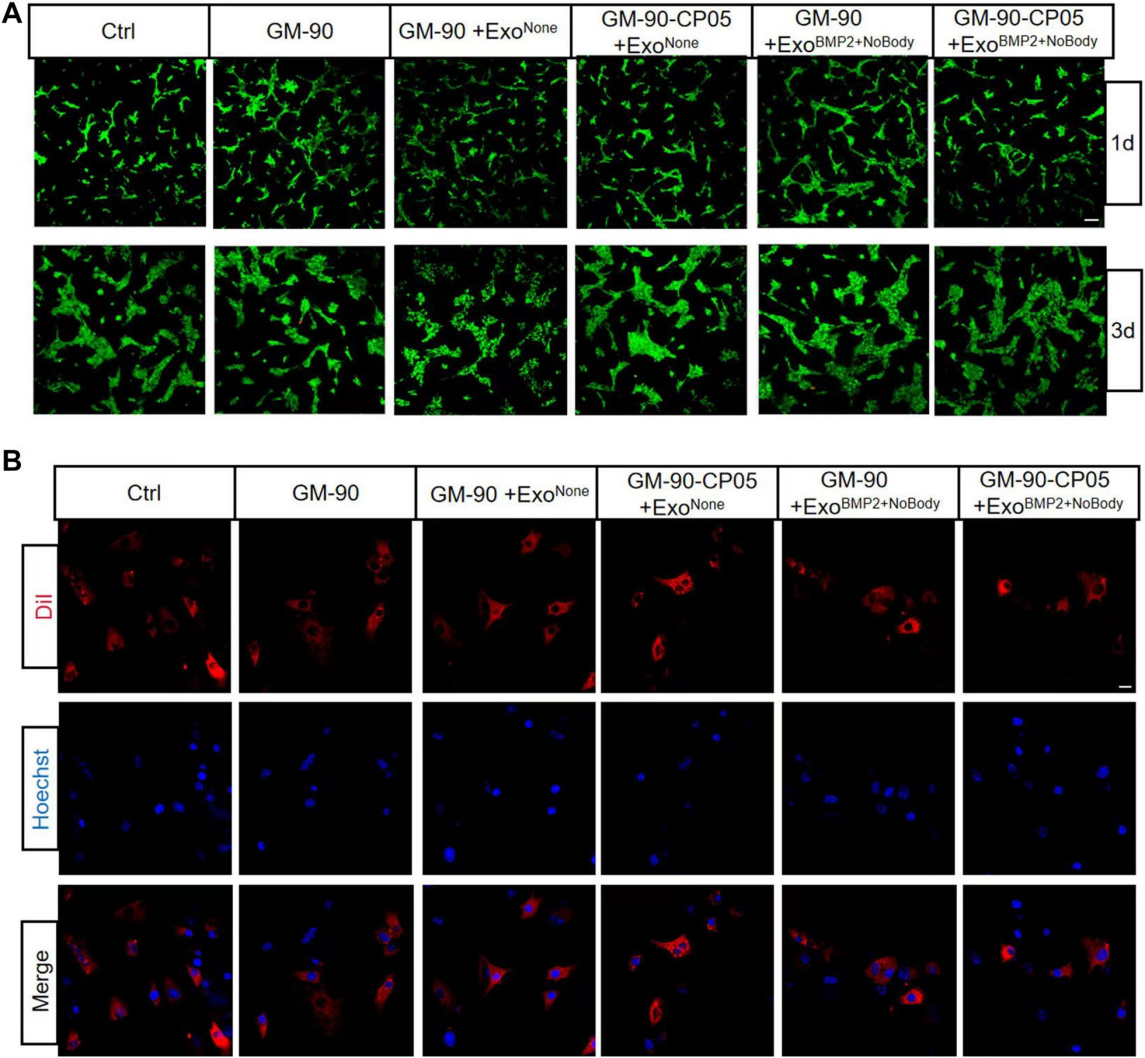
Biocompatibility of GelMA-CP05 hydrogel with rBMSCs. **A** Live/dead staining of BMSCs on the hydrogel after days 1 and 3 of incubation. Scale bar = 100 µm. **B** Internalization of DiI-labelled EXOs encapsulated in hydrogel into BMSCs was visualized using confocal images. Scale bar = 10 μm

**Fig. 6:**
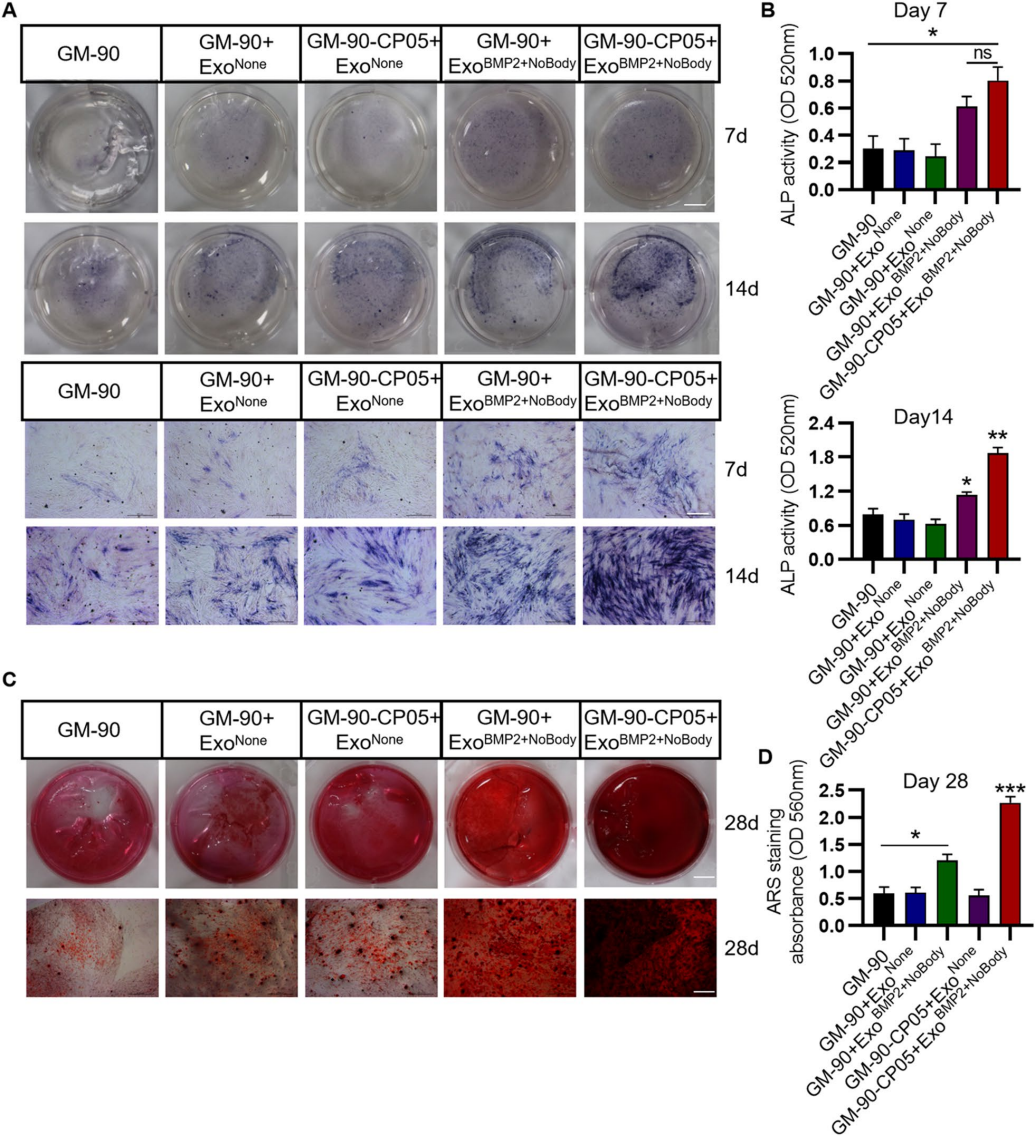
Enhanced osteogenesis mediated by GM-90-CP05 + Exo^BMP2+NoBody^. ALP expression (using ALP staining) (**A**) and activity (**B**) were assessed on days 7 and 14, respectively. Scale bar = 1 cm (top); scale bar = 500 µm (below). Mineralization of rBMSCs was detected using Alizarin red staining (**C**) and quantified (**D**) on day 28. Scale bar = 1 cm (top); scale bar = 500 µm (below).

BMSCs were seeded on different types of hydrogels, cultured for 1 and 3 days for live and dead cell staining, and cultured for 1, 3, 5, and 7 days for CCK8 detection. There was no significant difference in the biocompatibility and cell proliferation in the hydrogels among the groups. In other words, the viability of cells seeded on GM-90-CP05 hydrogel combined with Exo^BMP2+NoBody^ was comparable with that of the control group (Fig. 5A; Additional file 1: Fig. S3). After 2 days of culture, confocal images showed that DiI-labelled exosomes could be released from different types of hydrogels and taken up by BMSCs (Fig. 5B), which was consistent with the results of in vitro co-culture without hydrogel. In the presence of Exo^BMP2+NoBody^, BMSCs seeded on GM-90-CP05 hydrogel exhibited increased osteogenesis and elevated ALP expression and mineralization levels than the control group (Fig. 6A–D). It was also confirmed that the GM-90-CP05 hydrogel could maintain the osteogenic activity of Exo^BMP2+NoBody^ for a long duration.

### In vivo GM-90-CP05 hydrogel delivery of Exo^BMP2+NoBody^ promotes calvarial bone healing

**Fig. 7:**
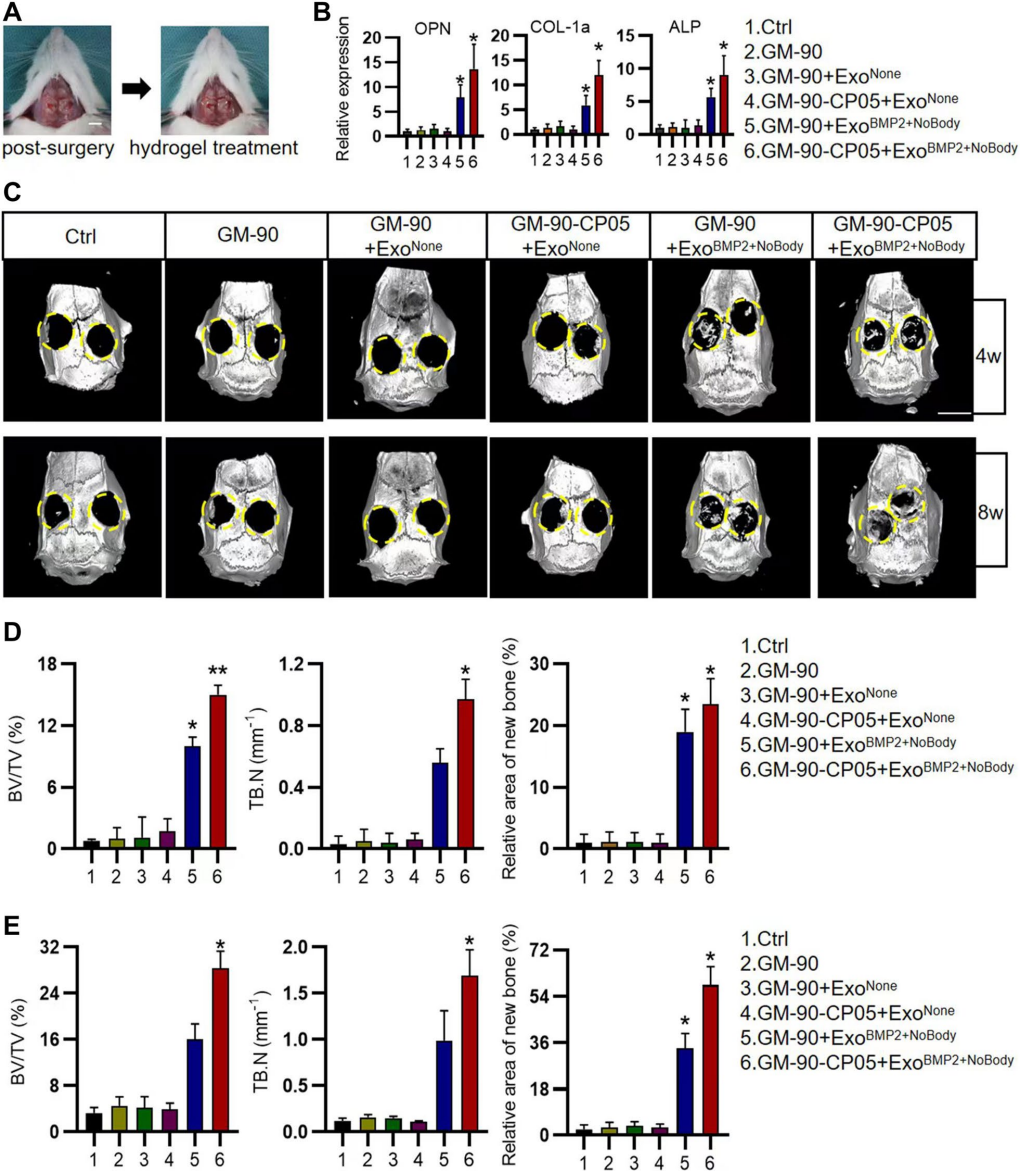
GM-90-CP05 + ExoBMP2 + NoBody promotes calvarial bone repair in vivo. **A** Photo of rat cranial defect model as well as the model after hydrogel treatment. Scale bar = 5 cm. **B** Expression of osteogenic related genes (*OPN, ALP* and *COL-1a*) was measured using qRT-PCR assay on day7. **C** Micro-CT images of rat calvarial bone regeneration at weeks 4 and 8. Scale bar = 5 cm. Quantitative analyses of new bone area, bone volume/tissue volume (BV/TV), trabecular number (Tb.N) (n = 6 per group) at weeks 4 (**D**) and 8 (**E**)

**Fig. 8:**
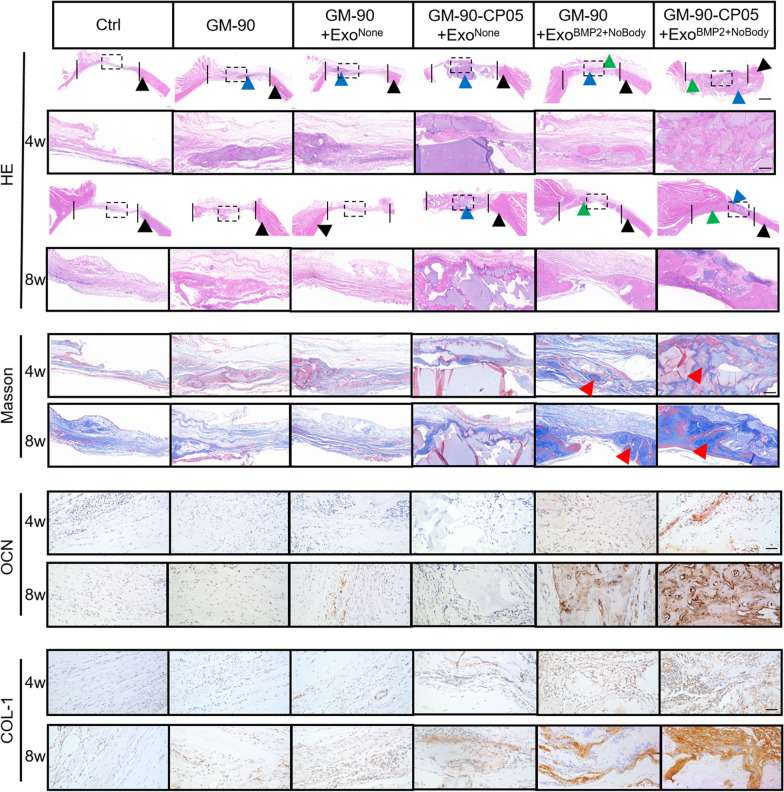
GM-90-CP05 + ExoBMP2 + NoBody-mediated delayed degradation and sustained release of exosomes induced new bone deposition in the defect. H&E stain: The vertical dashed lines indicate the relative original defect area. Green arrowheads indicate new bone tissues. Blue arrowheads indicate the hydrogel. Black arrowheads indicate native bone. The dashed box (black) indicates high magnification of the image below. Scale bar = 1 cm (top); scale bar = 200 µm (below). Masson stain: Red arrowheads indicate new bone tissues. Scale bar = 200 µm. OCN and COL-1 immunohistochemical stain: Scale bar = 100 µm

Based on the in vitro 2D and 3D results, we further investigated the efficiency of Exo^BMP2+NoBody^ in promoting bone repair in a rat calvarial defect model. Exo^BMP2+NoBody^ was loaded into GM-90-CP05 hydrogel and then injected into critical size calvarial defects in SD rats (Fig. 7A). After 7 days, the expression of major osteogenic genes such as *ALP, OPN* and *COL1a* was significantly increased in GM-90-CP05+EXO^BMP2+NoBody^ (P＜0.05) (Fig. 7B). Micro-CT analysis showed that the implant of GM-90-CP05 + Exo^BMP2+NoBody^ promoted significant bone healing compared with that in the remaining five groups. In the 8th week, in the GM-90-CP05 + Exo^BMP2+NoBody^ group, the defective bone was healed in a large area. Compared with the other four groups, except for GM-90-CP05 + Exo^BMP2+NoBody^, GM-90 + Exo^BMP2+NoBody^ also had obvious bone regeneration, but the bone healing was slower than that in GelMA + Exo^BMP2+NoBody^. In the 8th week, the gap of bone repair rate between GM-90 + Exo^BMP2+NoBody^ and GM-90-CP05 + Exo^BMP2+NoBody^ further widened, indicating that GM-90-CP05 + Exo^BMP2+NoBody^ had better sustained bone repair ability. New bone area, bone volume/tissue volume (BV/TV), and trabecular number (Tb. N) measurements indicated similar trends, with the largest values found in the GM-90-CP05 + Exo^BMP2+NoBody^ group (P < 0.05) (Fig. 7C, D). The substantial bone healing of the GM-90-CP05 + Exo^BMP2+NoBody^ treatment group was also confirmed by histological and immunohistochemical analyses (Fig. 8). Haematoxylin and eosin staining showed that in the GM-90-CP05 + Exo^BMP2+NoBody^ treatment group, new lamellar bone formed, almost bridging the defective area 8 weeks after surgery, and the hydrogel was still not completely degraded. Although no obvious bone regeneration in GM-90-CP05 + Exo^None^ was observed, the hydrogel in the GM-90-CP05 + Exo^None^ group degraded slowly compared with that in GM-90, GM-90 + Exo^None^ and GM-90/CP05 + Exo^None^. GM-90 + Exo^BMP2+NoBody^ had a certain degree of bone regeneration, but the hydrogel was completely degraded at 8 weeks, and the bone regeneration process was significantly slower than that of GM-90-CP05 + Exo^BMP2+NoBody^. Likewise, a substantial amount of new bone was present in the GM-90-CP05 + Exo^BMP2+NoBody^-treated calvarial defect as detected using Masson staining when compared to that in the other treatment groups. No obvious bone formation was detected in the defective areas in the GM-90, GM-90 + Exo^None^, GM-90/CP05 + Exo^None^, and GM-90-CP05 + Exo^None^ groups. OCN and type I collagen are proteins associated with osteoblast and matrix mineralisation, as shown by immunohistochemical staining. The expression of OCN and COL-1a was markedly elevated in GM-90 + Exo^BMP2+NoBody^- and GM-90-CP05 + Exo^BMP2+NoBody^-treated defects compared with that in the remaining four groups. This indicates an increased degree of osteoblast differentiation and mineral isation in these treatment groups. In the 8^th^ week of treatment, the expression of OCN and COL-1a in Exo^BMP2+NoBody^ was considerably higher than that in GM-90 + Exo^BMP2+NoBody^, indicating that GM-90-CP05 + Exo^BMP2+NoBody^ played a persistent role in long-term bone repair. Taken together, these in vivo experiments demonstrate that delivery of Exo^BMP2+NoBody^ via injection of modified GelMA hydrogel induces robust and durable bone repair in a rat model of non-healing calvarial defect.

## Discussion

In this study, specific Bmp2 mRNA-rich engineered exosomes were successfully obtained by co-transfection of NoBody and BMP2 artificial plasmids into 293T cells. Allyl-L-glycine-modified CP05 could be covalently bound to exosomes and GelMA, and GelMA-CP05 was used as a bioabsorbable scaffold for engineering exosomes to achieve sustained release of engineered exosomes. Finally, the osteogenesis of stem cells in vitro and in vivo was confirmed.

Two artificial plasmids were used to design engineered exosomes by co-transfection to achieve efficient, safe, and lasting bone regeneration therapy. Our study confirmed that engineered exosomes with high yield and enriched in BMP2 can be obtained by co-transfection of 293T cells with BMP2 and NoBody artificial plasmids. As described by Tutucci et al., the MS2 phage coat protein (MCP), which is in the NoBody sequence, specifically recognises and binds to the MS2-binding site (MBS) in the BMP2 sequence and protects the RNA from being efficiently degraded [31]. With the binding of MCP and MS2, NoBody recognises and binds to the 5′ cap of the target *Bmp2* mRNA in the cell, and the *Bmp2* mRNA entering the translation phase markedly decreases. It showed that *Bmp2* mRNA is excreted out of the cell through the exosomes pathway, yielding engineered exosomes abundant in mRNA. NoBody acts as promoter to some extent mainly due to the increased loading efficiency. When the engineered exosomes are ingested by the BMSCs, the mRNA passes through the IRES in the sequence [32] and enters into the autonomous translation process expressing BMP2; the experimental results confirmed that NoBody does not affect the *Bmp2* mRNA translation. Exosomes obtained by this pathway contained approximately three-fold more *Bmp2* mRNA than those transfected with the BMP2 plasmid alone, and the intracellular BMP2 expression was also correspondingly higher after co-culture with recipient cells.

In bone-related diseases, such as periodontitis, fractures, tumours, and surgical trauma, regeneration of defective bone is a crucial therapeutic goal [33]. Recombinant human bone morphogenetic protein-2 (rhBMP-2) has been approved for use in the United States and Europe, but the clinical efficacy of rhBMP-2 has been disappointing because of insufficient delivery systems. Although collagen has been approved by the U.S. Food and Drug Administration for BMP-2 delivery systems, the financial cost is greatly increased because of the need to apply large amounts of recombinant protein to overcome the deficiencies of collagen sponges as protein delivery scaffolds [34]. Additionally, there are problems such as immunogenicity, low binding rate, poor retention rate, poor release control, and ectopic bone growth complications caused by BMP-2 overflow [35]. Various approaches have been developed to improve protein delivery, including the use of smart scaffolds. Unfortunately, none of these studies made it into clinical trials [36, 37]. Gene therapy clinical trials in recent years have shown significant therapeutic effects and a good safety profile and will therefore be a potential alternative therapy option in the future [38]. The rapid degradation and predictable pharmacokinetics and pharmacodynamics of RNAs make them safer and more economical than DNA for use in therapy [39]. RNAs for medical applications are in the form of mRNA, siRNA, miRNA, and ASO. Among them, mRNA can be used in therapeutics, diagnostics, and vaccines [40]. In therapeutics, mRNA translation produces therapeutic proteins to replace defective or missing proteins, and can also serve as therapeutic targets for ASOs, siRNAs, miRNAs, aptamers, and inhibitory tRNAs. In diagnostics, mRNA dynamic detection technology can be used for the diagnosis or prognostic evaluation of various diseases. In vaccines, mRNA translation can serve as an antigenic target for the immune system, such as the spike glycoprotein of SARS-CoV-2 in the Covid-19 mRNA vaccine [41]. At present, mRNA therapy has made great progress in cardiovascular diseases [42], respiratory diseases [43], metabolic diseases [44], infectious diseases [45], autoimmune diseases [46], and tumours [47]. Compared with other biomolecules, RNA molecules are hydrophilic and negatively charged, cannot diffuse through cell membranes, are unstable and easily degraded, and may trigger immunogenicity in vivo. However, these problems can be alleviated by chemically modifying the RNA. Excellent vectors can better protect therapeutic genes and safely deliver them into specific cells. Therefore, vector improvement is one of the key links in the development and breakthrough of gene therapy, which is essential for overcoming the physiological barriers of systemic administration, which is the basis for gene therapy applications [48].

Lipid carriers have become the main RNA delivery vehicles [48]. However, because lipid nanoparticles may be toxic to cells and stimulate the release of systemic inflammatory cytokines, there is increasing interest in natural transporters such as exosomes [49]. Nearly 20 years after liposomes were first created, scientists have discovered exosomes in most eukaryotic cells, which may play important roles in intercellular communication and signalling, as well as in the transport of proteins, lipids, and nucleic acids between cells. They are thought to be involved in important physiological processes, such as the regulation of intercellular communication and signalling, and the transmission of macromolecules between cells [50]. Compared to other nanocarriers, exosomes act as natural transporters [51] that are neither toxic to cells nor do they elicit an immune response. Further, they do not lead to deleterious accumulation of therapeutic RNAs in the liver. Additionally, exosomes themselves contain exosomal RNAs in the form of mRNA, miRNA, siRNA, and lncRNA [52]. They can be packaged and transported to recipient cells through the exosome pathway, so they are considered good candidates for mRNA delivery [53]. In this study, exosomes were used as mRNA delivery vehicles for bone regeneration therapy. First, exosomes can circumvent endocytosis and escape rapid clearance by cells of the reticuloendothelial system. Second, internalization of exosomes by target cells promotes mRNA exchange [54–56], resulting in higher delivery efficiency, which in turn affects target cells involved in bone tissue repair. GelMA acts as a local delivery system of exosomes and new bone scaffold, which has good cell compatibility, and CP05 has specific exosome anchoring characteristics. This study shows that the covalent cross-linking of GelMA and CP05 can greatly delay the degradation time of hydrogel, enrich and slowly release engineered exosomes, and achieves sustained release and safe treatment of defective areas.

In summary, this study investigated gene therapy using exosomes as a vector, initiated a new RNA therapy, verified the NoBody mechanism of action, and applied it to the development of engineered exosomes. Combined with a CP05 modified hydrogel, we achieved a long-term and safe method for bone regeneration. The present results provide new strategies for the development and future design of engineered. The optimal proportion of GM90/CP05/Exo incorporation, the improved mechanical properties of the hydrogel, and molecular mechanisms related to osteogenesis will require further investigation and exploration in future studies.

## Conclusion

Our study demonstrated that expressional inhibition of the target mRNA in the parental cell leads to the enrichment of target-mRNA-rich exosomes, which can then be transported to the target cell to produce therapeutic proteins to treat disease. Through this strategy, we designed the BMP2 and NoBody plasmids and obtained the engineered exosomes enriched in *Bmp2* mRNA. This developed exosome has a remarkable effect on bone regeneration. Engineered exosomes bind to the hydrogel scaffold with modified CP05. This combination effectively increases the continuous release of exosomes and promotes osteogenesis of critical bone defects. Therefore, our current study presents a new strategy for bone regeneration therapy, namely, RNA therapy techniques with exosomes as carriers.

## Supplementary Information

**Additional file 1: Table S1.** Sequence composition of The artificial plasmid. **Table S2.** Sequences of PCR primers. **Figure S1. **Formulas for the chemical reaction of the (allyl-L-glycine)-CP05 with methylamide. **Figure S2.** Degradation experiment of hydrogel detected at sixty-hour intervals. **Figure S3.** Proliferation of rBMSCs cultured directly on the different hydrogels detected by using the CCK-8 kit (n = 3). **Figure S4.** 3D confocal images of GM-90/CP05+Exo Combination Group GM-90-CP05/Exo at 50h. **Figure S5.** Fluorescence intensity of different types of hydrogels encapsulated DiI labeled exosomes at various time points. **Figure S6. **The whole membrane of BMP2 for Western Blotting. **Figure S7. **The degradation time of GM-90-CP05+Exo gel in rats.

## Data Availability

The authors declare that the main data supporting the findings of this study are included in this published article and its additional file information.

## Abbreviations

BMP2: Bone morphogenetic protein-2
NoBody: Non-annotated P-body dissociating polypeptide
BMSCs: Bone marrow stromal cells
BSA: Bovine serum albumin
DMEM: Dulbecco’s modified Eagle’s medium
FBS: Foetal bovine serum
FDA: Food and Drug Administration
IRES: Intrinsic ribosomal entry site
NTA: : Nanoparticle tracking analysis
PBS: Phosphate-buffered saline
SD: Sprague Dawley
TEM: Transmission electron microscopy
