# Supplementary material for: Hydrogel armed with Bmp2 mRNA-enriched exosomes enhances bone regeneration
Source: J Nanobiotechnology. 2023 Apr 5;21:119. doi: 10.1186/s12951-023-01871-w (PMC10075167; doi:10.1186/s12951-023-01871-w)
Supplement: Supplementary file 1 — Additional file 1: Table S1. Sequence composition of The artificial plasmid. Table S2. Sequences of PCR primers. Figure S1. Formulas for the chemical reaction of the (allyl-L-glycine)-CP05 with methylamide. Figure S2. Degradation experiment of hydrogel detected at sixty-hour intervals. Figure S3. Proliferation of rBMSCs cultured directly on the different hydrogels detected by using the CCK-8 kit (n = 3). Figure S4. 3D confocal images of GM-90/CP05+Exo Combination Group GM-90-CP05/Exo at 50h. Figure S5. Fluorescence intensity of different types of hydrogels encapsulated DiI labeled exosomes at various time points. Figure S6. The whole membrane of BMP2 for Western Blotting. Figure S7. The degradation time of GM-90-CP05+Exo gel in rats. [file 12951_2023_1871_MOESM1_ESM.docx]

**Supplementary Information**

**Hydrogel armed with *Bmp2* mRNA-enriched exosomes enhances bone regeneration**

Zhujun Yang^1,2,3^, Xuejian Li^2^, Xueqi Gan^4^, Mengying Wei^3^, Chunbao Wang^5^, Guodong Yang^3^, Yimin Zhao^2,*^, Zhuoli Zhu^4,**^, Zhongshan Wang^2,***^

**Table**

Table S1. Sequence composition of The artificial plasmid.

|  | **Sequence** |
| --- | --- |
| MS2 | acatgaggatcacccatgt |
| Linker | ctgcagggcctagcaagttaaaataaggctagtccgttatcaacttggcc |
| IRES | acgttactggccgaagccgcttggaataaggccggtgtgcgtttgtctatatgttattttccaccatattgccgtcttttggcaatgtgagggcccggaaacctggccctgtcttcttgacgagcattcctaggggtctttcccctctcgccaaaggaatgcaaggtctgttgaatgtcgtgaaggaagcagttcctctggaagcttcttgaagacaaacaacgtctgtagcgaccctttgcaggcagcggaaccccccacctggcgacaggtgcctctgcggccaaaagccacgtgtataagatacacctgcaaaggcggcacaaccccagtgccacgttgtgagttggatagttgtggaaagagtcaaatggctctcctcaagcgtattcaacaaggggctgaaggatgcccagaaggtaccccattgtatgggatctgatctggggcctcggtgcacatgctttacatgtgtttagtcgaggttaaaaaacgtctaggccccccgaaccacggggacgtggttttcctttgaaaaacacgatgataata |
| rBMP2 | atggtggccgggacccgctgtcttctagtgttgctgcttccccaggtcctcctgggcggcgcggccggcctcattccggagctgggccgcaagaagttcgccggggcatccggccgccccttgtcccggccttcggacgacgtcctcagcgagtttgagttgaggctgctcagcatgtttggcctgaagcagagacccacccccagcaaggacgtcgtggtgcccccctatatgctcgacctgtaccgccggcactcgggccagccaggagcgcccgccccagaccaccggctggagagggcagccagccgcgccaacaccgtgcgcagcttccatcacgaagaagccatcgaggaacttccagaaatgagtgggaaaacgtcccgacgcttcttcttcaatttaagttctgtccctactgatgagtttctcacatctgcggagctccagatttttcgggaacaaatgcaggaagctttgggaaatagtagtttccagcaccgaattaatatttatgaaattataaagcctgccacagccagctcaaaatttcctgtgaccagactattggacaccaggttagtgactcagaacacaagtcagtgggagagctttgatgtcaccccggctgtgatgcgatggacagcacagggacacaccaaccatgggtttgtggtggaagtggcccacttagaggagaagccaggtgtctccaagagacatgtgaggattagcaggtctttgcaccaagatgaacacagctggtctcaggtaagaccactgctagtgacttttggccacgacggaaaaggacatccactccacaaacgagaaaagcgtcaagccaaacacaaacagcggaagcgtcttaagtccagctgcaaaaggcaccctttgtatgtggacttcagtgatgtggggtggaatgactggatcgtggcccctccaggctatcatgccttttactgccatggggaatgtccttttcccctggctgatcacctgaactccaccaaccatgccatagtgcagactctggtaaactctgtgaattccaaaatccctaaggcatgctgtgtccccactgagcttagcgcaatctccatgttgtacctagatgaaaacgaaaaggttgtgctaaaaaactatcaggacatggttgtggagggttgcgggtgtcgctag |
| Flag | gactacaaggatgacgacgataag |
| NoBody | atgggagaccaaccttgtgcctccgggagatccactctcccacctggaaacgcacgggaagccaagcctccaaaaaagcgctgcctcctcgctccgcgttgggattatccggaaggaactcccaacggaggtagtaccactctaccctccgcacctcctcctgcatcagccggcctgaagtcgcaccctcctcctccggagaag |
| MCP | ATGGCTTCAAACTTTACTCAGTTCGTGCTCGTGGACAATGGTGGGACAGGGGATGTGACAGTGGCTCCTTCTAATTTCGCTAATGGGGTGGCAGAGTGGATCAGCTCCAACTCACGGAGCCAGGCCTACAAGGTGACATGCAGCGTCAGGCAGTCTAGTGCCCAGAAGAGAAAGTATACCATCAAGGTGGAGGTCCCCAAAGTGGCTACCCAGACAGTGGGCGGAGTCGAACTGCCTGTCGCCGCTTGGAGGTCCTACCTGAACATGGAGCTCACTATCCCAATTTTCGCTACCAATTCTGACTGTGAACTCATCGTGAAGGCAATGCAGGGGCTCCTCAAAGACGGTAATCCTATCCCTTCCGCCATCGCCGCTAACTCAGGTATCTACTAG |

Table S2. Sequences of PCR primers.

| **Gene** | **Primer sequence** |
| --- | --- |
| rGAPDH | F：GGCACAGTCAAGGCTGAGAATG  R：ATGGTGGTGAAGACGCCAGTA |
| rBMP2 | F：ATGGGTTTGTGGTGGAAGTG  R：TTGGCTTGACGCTTTTCTCG |
| rColla Ia | F：AAGTCTCAAGATGGTGGCCG  R：TACTCTCCGCTCTTCCAGTCA |
| rOPN | F：GACGGCCGAGGTGATAGCTT  R：CATGGCTGGTCTTCCCGTTGC |
| rALP | F：CCTGCAGGATCGGAACGTCAATTA  R：TGAGTTGGTAAGGCAGGGTCC |

**Figure**

**
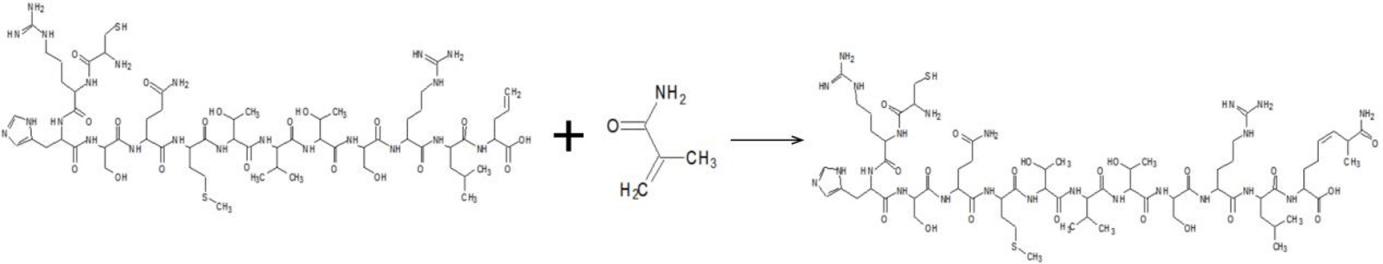
**

**Fig. S1** Formulas for the chemical reaction of the (allyl-L-glycine)-CP05 with methylamide

**
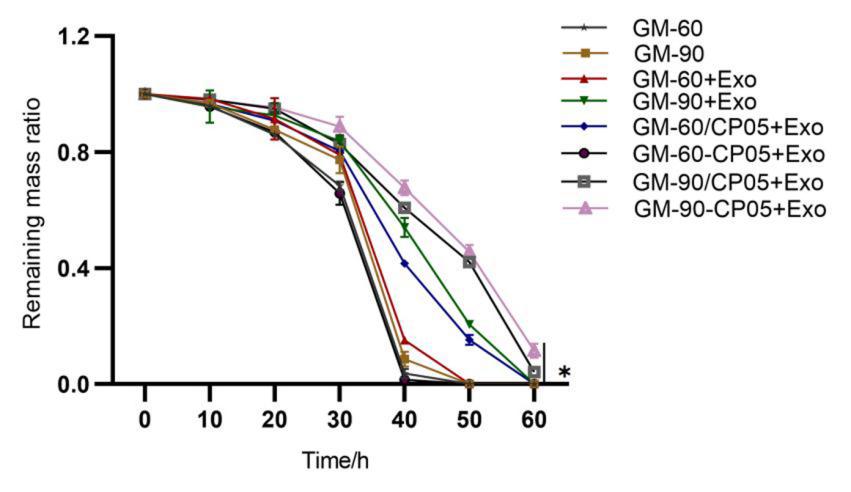
**

**Fig. S2** Degradation experiment of hydrogel detected at sixty-hour intervals.


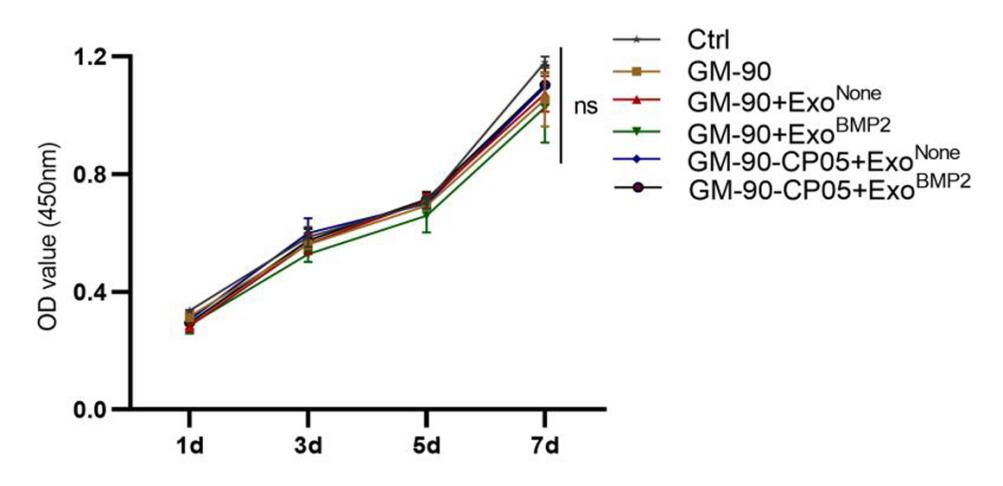


**Fig. S3** Proliferation of rBMSCs cultured directly on the different hydrogels detected by using the CCK-8 kit (n = 3).


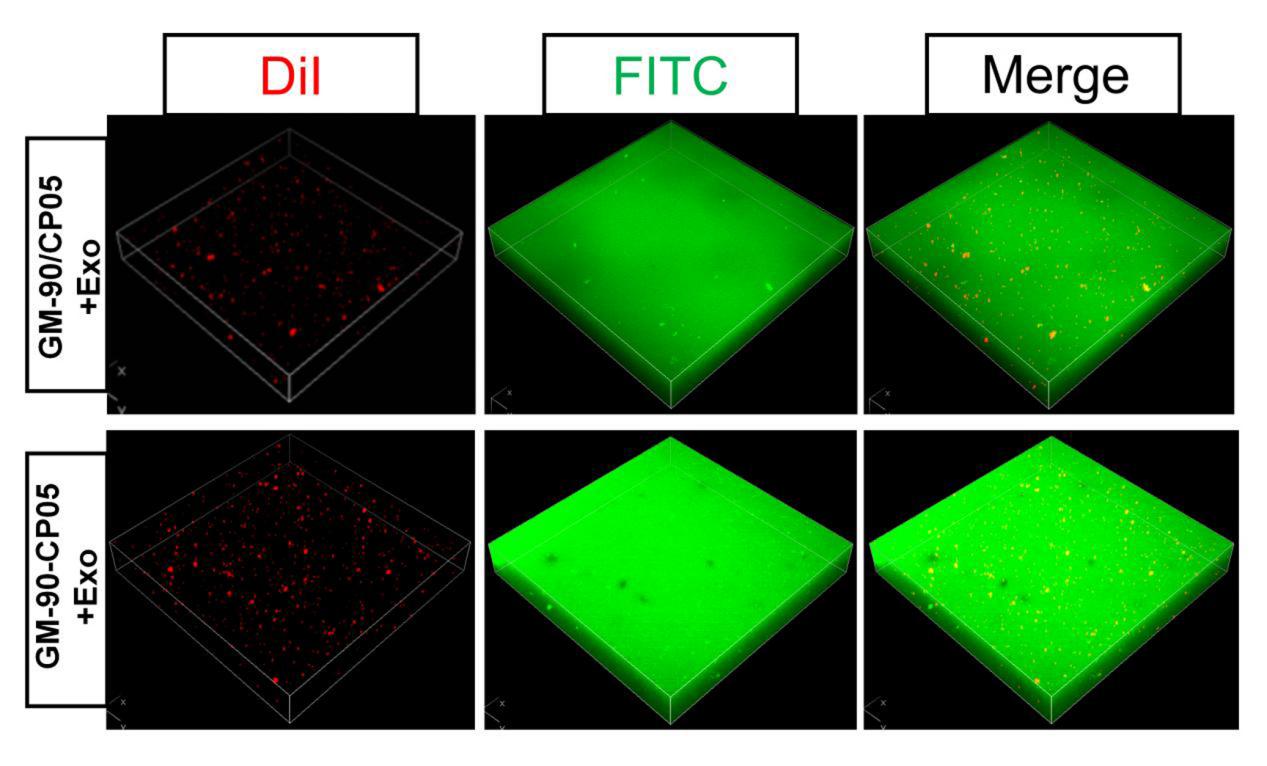


**Fig. S4** 3D confocal images of GM-90/CP05+Exo Combination Group GM-90-CP05/Exo at 50h


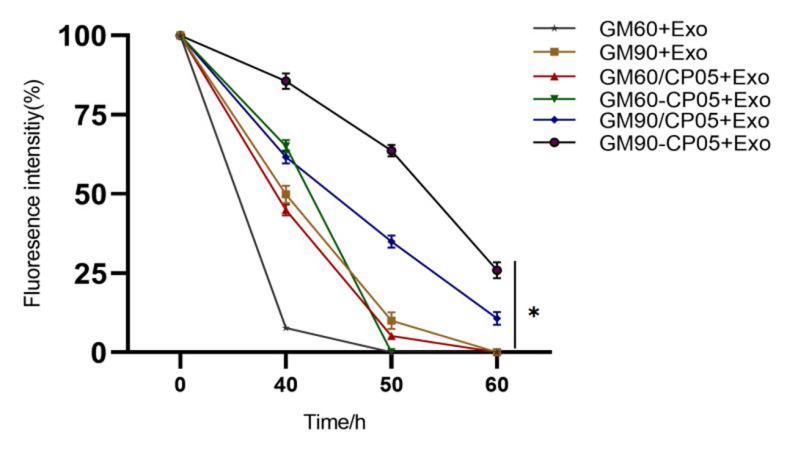


**Fig. S5** Fluorescence intensity of different types of hydrogels encapsulated DiI labeled exosomes at various time points


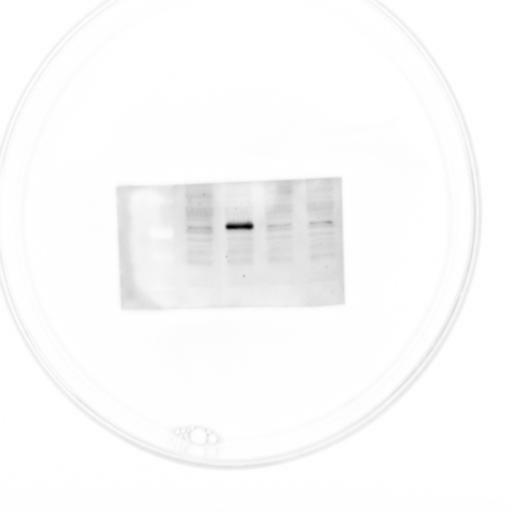

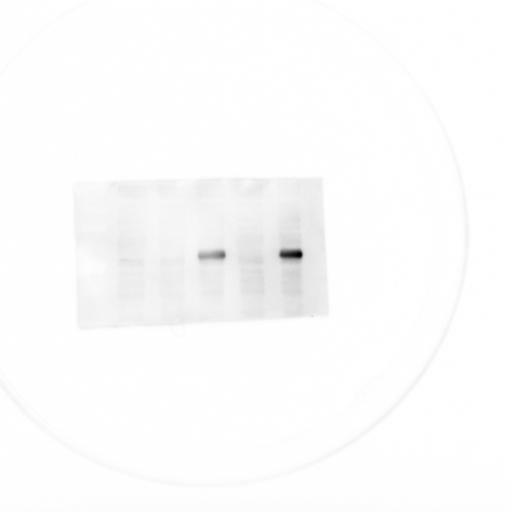


**Fig. S6** The whole membrane of BMP2 for Western Blotting


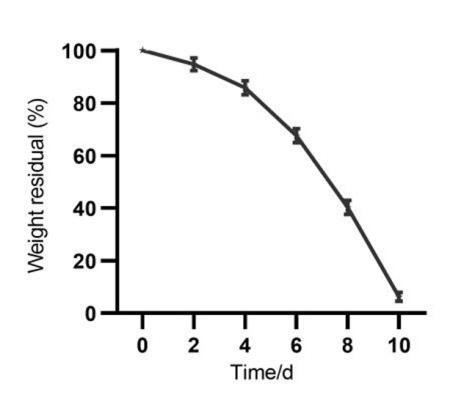


**Fig. S7** The degradation time of GM-90-CP05+Exo gel in rats.
